# Spontaneous Pneumomediastinum in a Young Adult: A Rare Presentation

**DOI:** 10.7759/cureus.8306

**Published:** 2020-05-26

**Authors:** Theakarajan Rajendran, Oseen Shaikh, Uday Kumbhar, Gopal Balasubramanian, Sandeep Bhattarai

**Affiliations:** 1 Surgery, Jawaharlal Institute of Postgraduate Medical Education and Research (JIPMER), Puducherry, IND

**Keywords:** spontaneous pneumomediastinum, subcutaneous emphysema

## Abstract

Spontaneous pneumomediastinum (SPM) refers to the presence of air in the mediastinum without any obvious cause. It is an uncommon condition occurring due to alveolar rupture as a result of increased intrathoracic pressure. It is commonly seen in young males, patients with known asthmatic disorders and pulmonary diseases. We report a rare case of SPM in a young healthy male without any significant past history. The patient was managed conservatively and discharged.

## Introduction

Pneumomediastinum or mediastinal emphysema is the presence of air in the mediastinum. It can be spontaneous or secondary following trauma, hollow organ perforation, iatrogenic injuries, and infections. Spontaneous pneumomediastinum (SPM) is defined as the presence of interstitial air in the mediastinum without any precipitating factors. SPM was first described by Hamman, hence it’s called Hamman's syndrome [1]. It is common among young males in the age group of 20 to 40 years, and has a benign course [2,3]. The classical triad of presentation includes retrosternal chest pain, dyspnoea, and subcutaneous emphysema [4]. It is important to distinguish pneumomediastinum from the conditions with similar clinical ﬁndings that require immediate treatment, such as cardiac tamponade, angina pectoris, dissecting aortic aneurysm, mediastinitis, and pulmonary embolism. We report a rare case of SPM in a young male who was diagnosed promptly and managed conservatively.

## Case presentation

A 22-year-old male nursing student presented to the emergency department with complaints of retrosternal pain and dyspnoea for one day. The patient also complained that he could feel some air passing through the tissues below the skin in the neck. The patient had a history of throat pain and intermittent cough for three days and a history of ingestion of hot water a day before the presentation. He denied any history of trauma to the neck or any strenuous activity over the past one week. His other medical history was unremarkable. He had no addictions and not undergone any surgeries in the past. Physical examination revealed a healthy-looking young man with tachypnoea. His pulse rate was 112 beats/min and blood pressure was 120/70 mmHg, his respiratory rate was 24/min. Crepitations were felt in the neck region extending up to the left nipple over the chest. The breath sounds and heart sounds were normal. Other systemic examinations were within normal limits.

**Figure 1:**
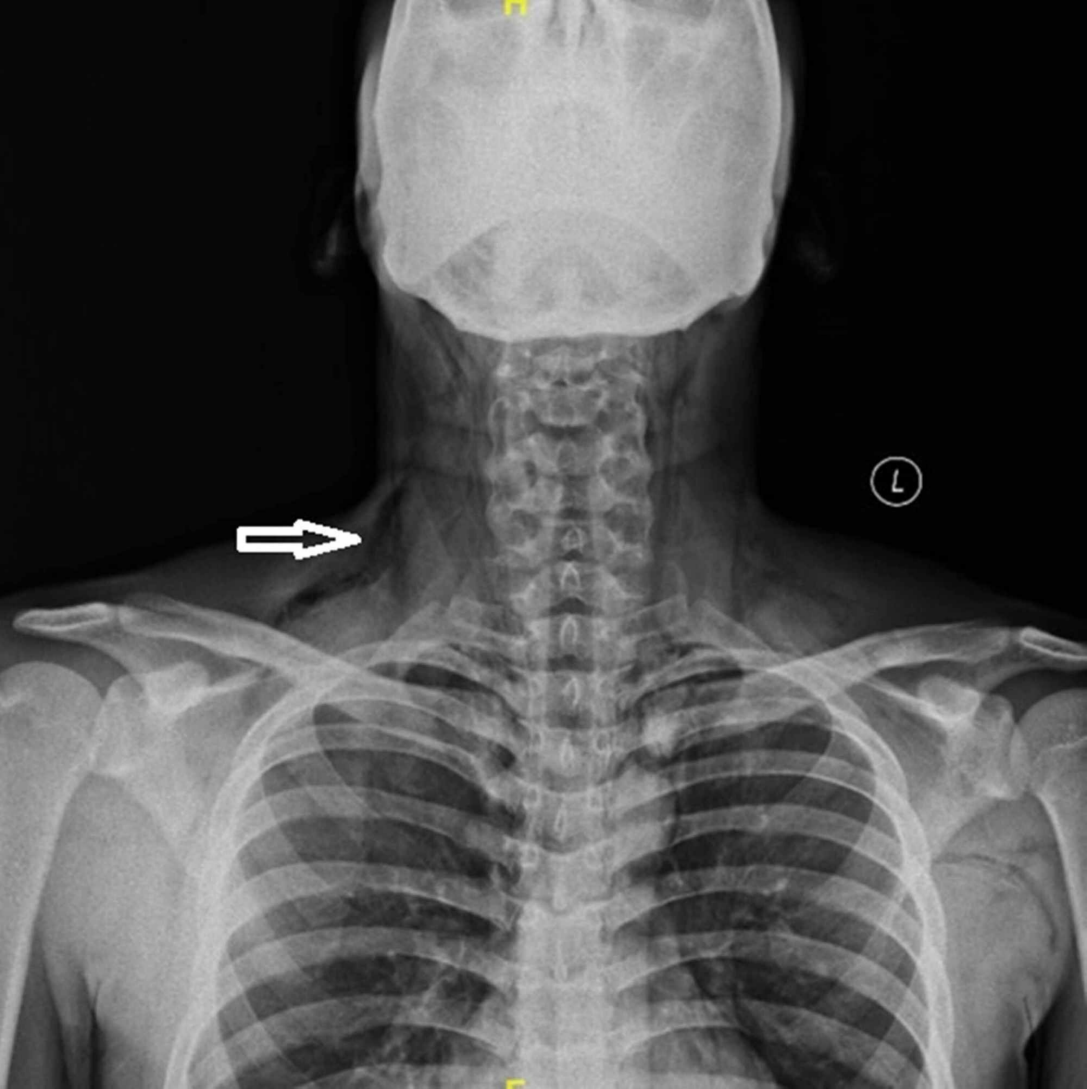
X-ray neck showing subcutaneous emphysema (arrow)

**Figure 2:**
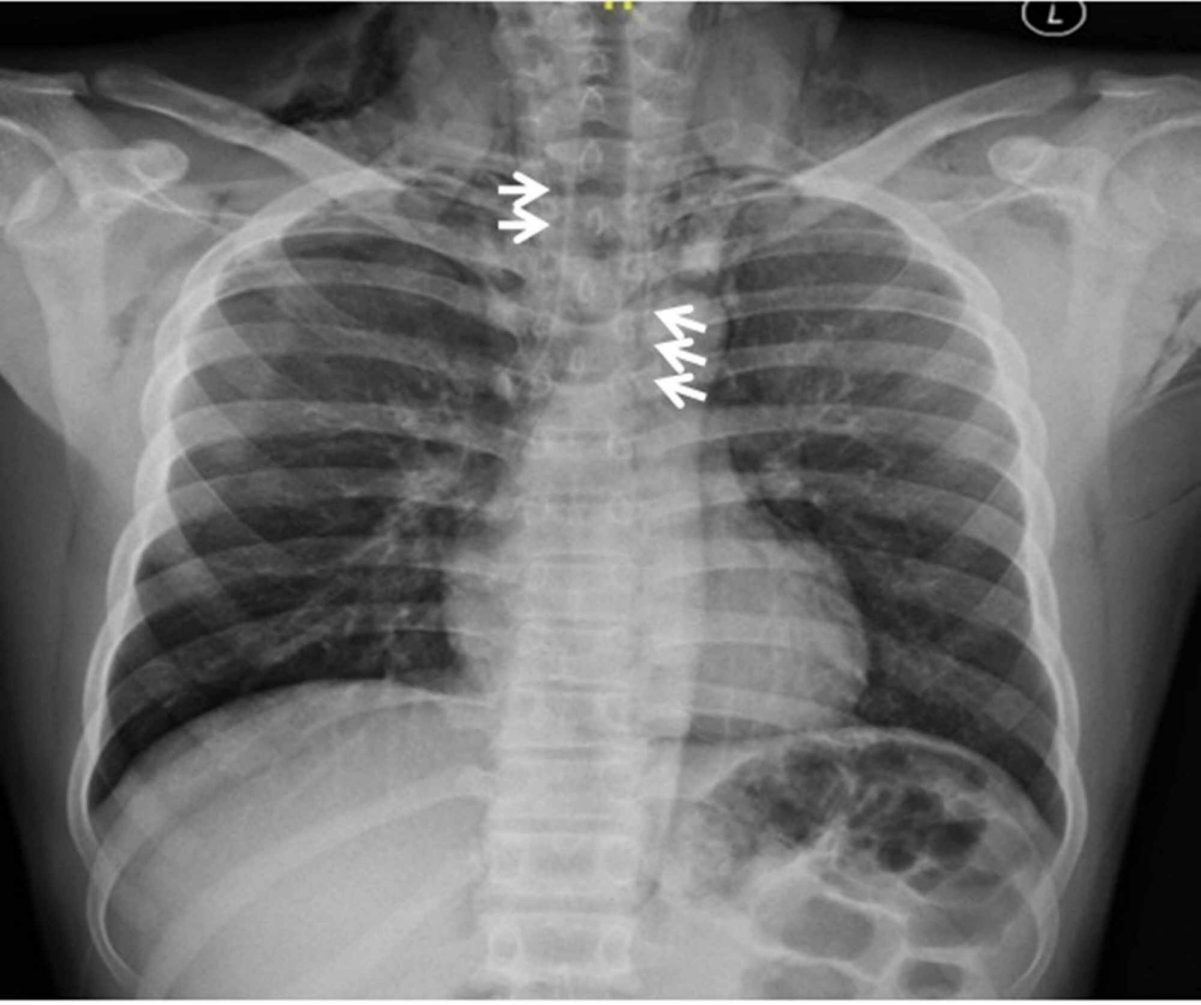
X-ray chest showing pneumomediastinum (arrows)

**Figure 3:**
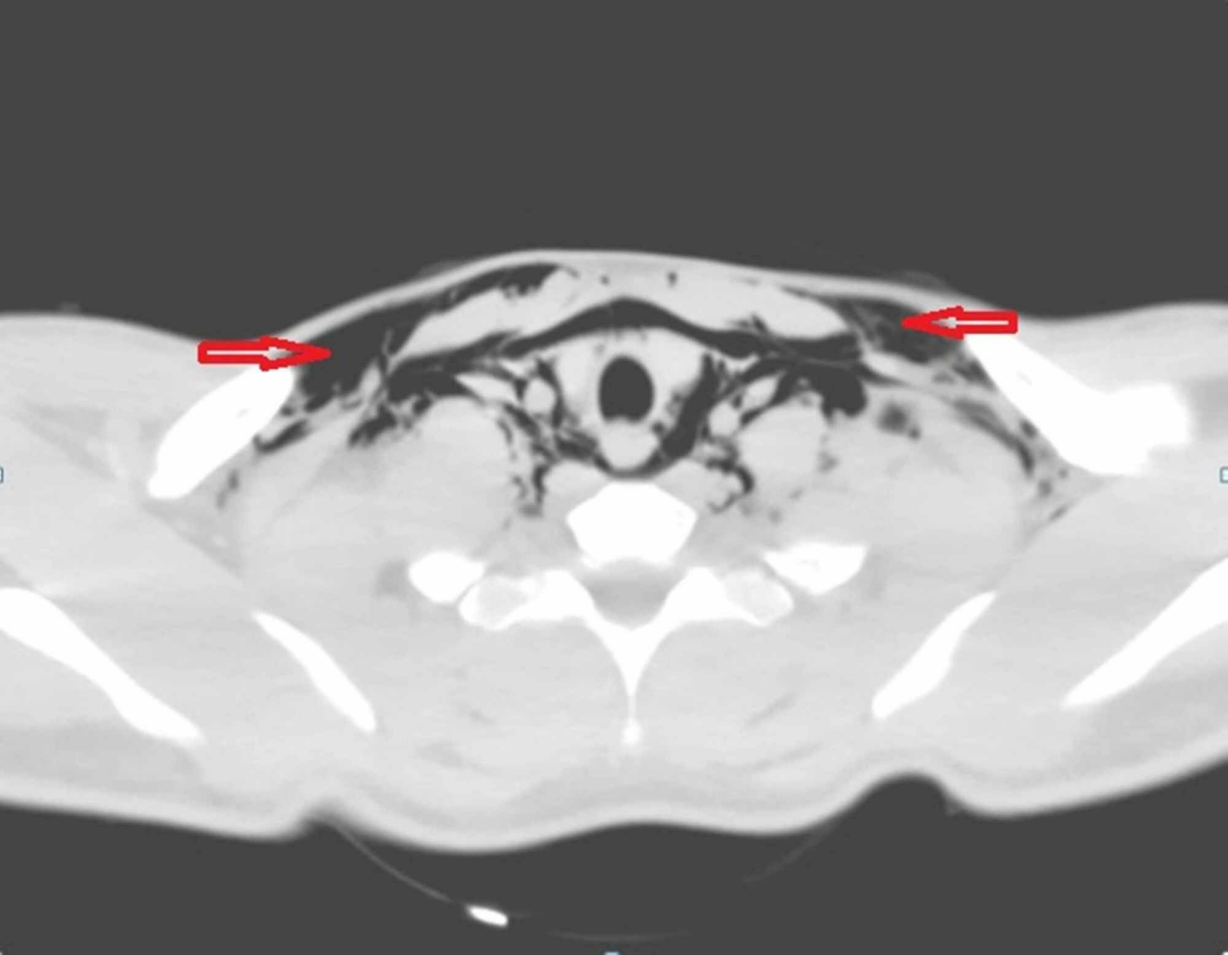
Non-contrast computed tomography (NCCT) of the neck showing subcutaneous emphysema (arrows)

**Figure 4:**
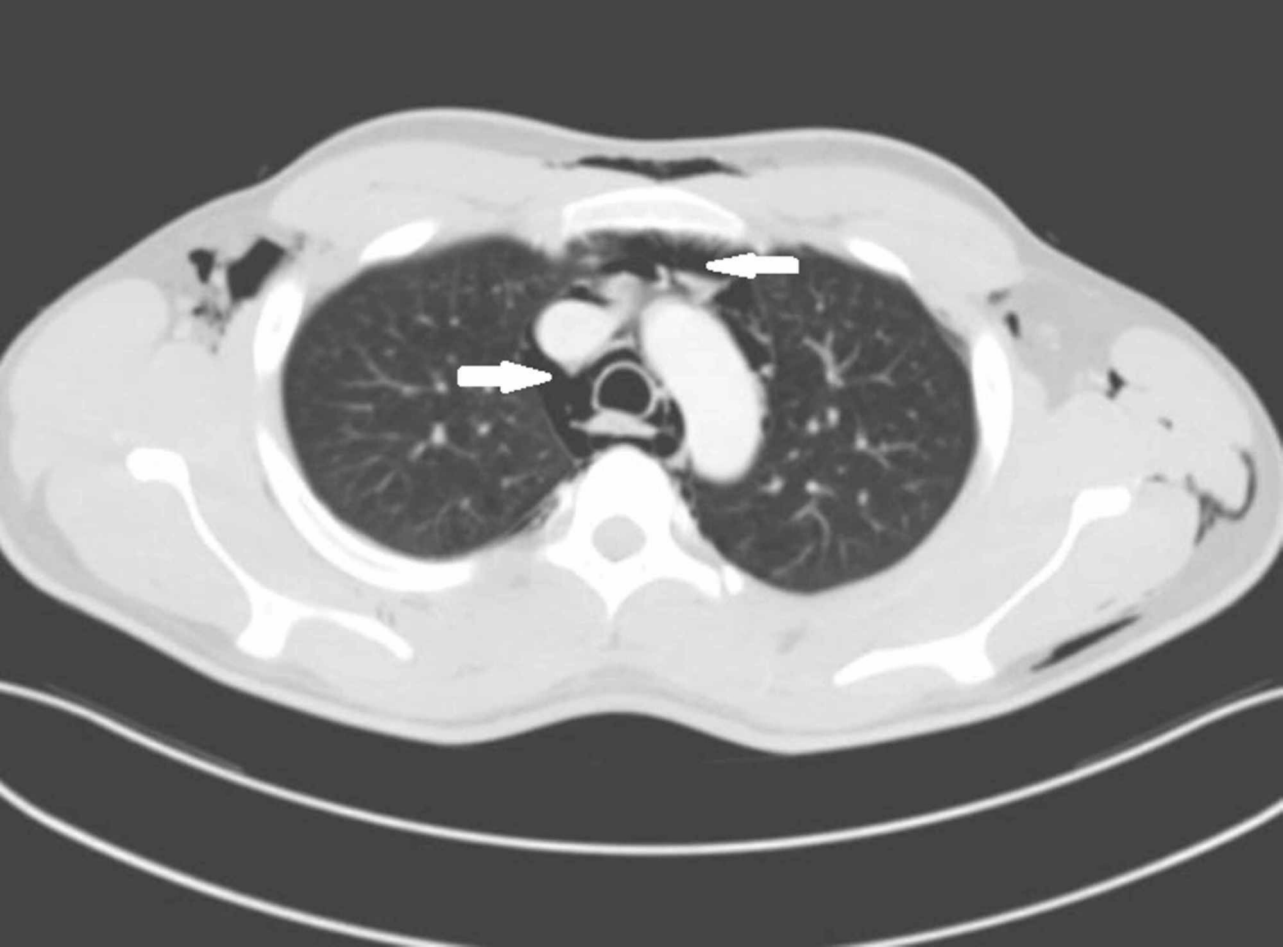
Contrast-enhanced computed tomography (CECT) of the chest showing pneumomediastinum (arrows)

**Figure 5:**
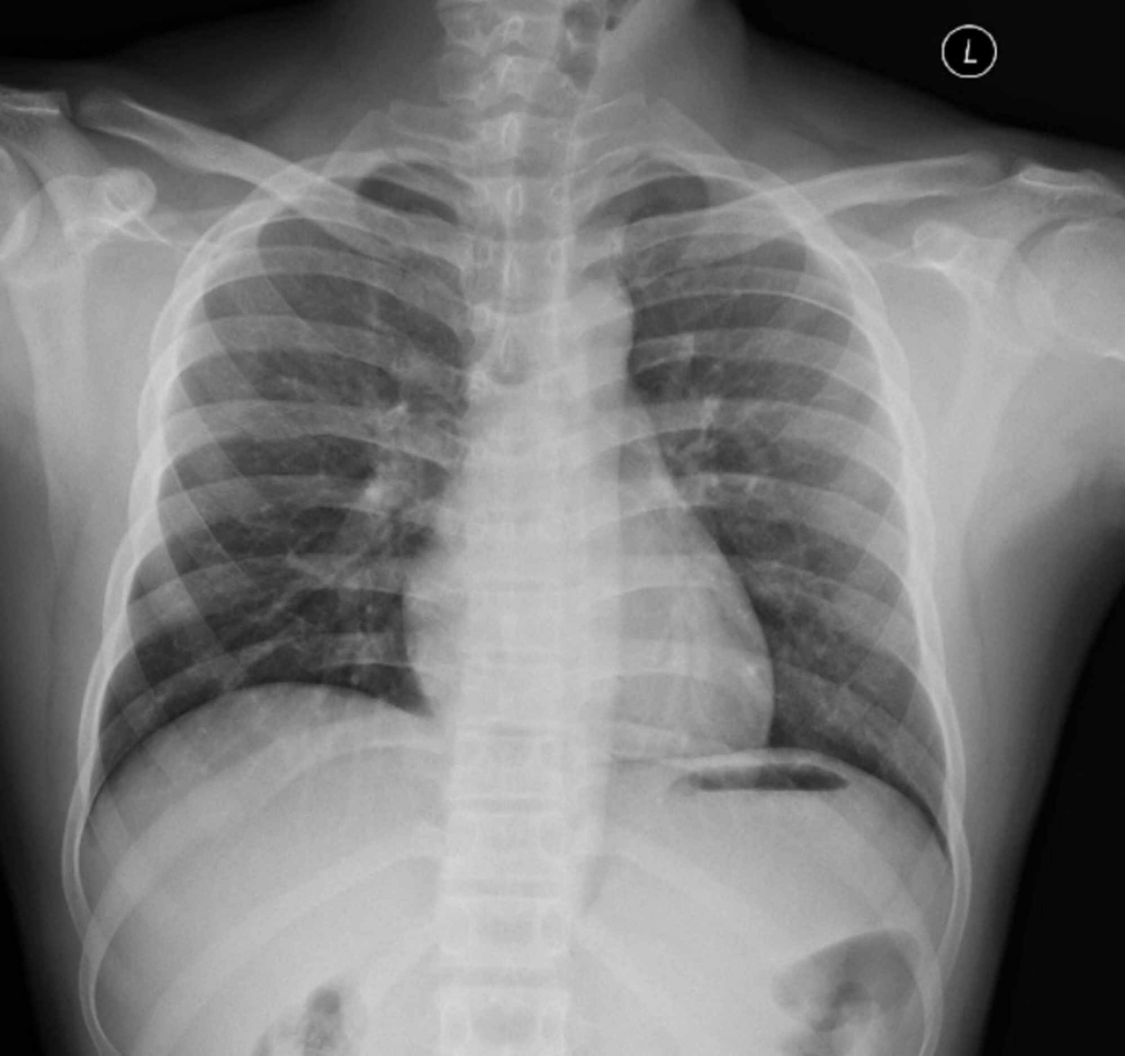
X-ray chest showing complete resolution of pneumomediastinum and subcutaneous emphysema

Laboratory values (complete blood count, renal function test, electrolytes) and arterial blood gas were within normal limits. His electrocardiogram (ECG) was normal. X-ray neck and chest showed pneumomediastinum and subcutaneous emphysema (Figures 1-2). Laryngoscopic examination did not reveal any mucosal lesion or foreign body. Computed tomographic scan of the head, neck and chest with oral gastrograffin was done which was suggestive of pneumomediastinum (Figures 3-4). There was no evidence of any oesophageal perforation, pneumothorax, pleural disease and lung pathology. Upper gastrointestinal endoscopy and bronchoscopy was also normal. The patient was admitted and managed conservatively. He was given oxygen by mask, analgesics, cough suppressants, and steroid nebulisation. His symptoms settled with conservative management in three days and hence the patient was discharged. The patient was followed up for six months and there was no recurrence of symptoms. Chest X-ray was repeated after three and six months and there was no evidence of pneumomediastinum (Figure 5). 

## Discussion

SPM is a rare entity which was first described by Louis Virgil Hamman in 1939 [1]. Secondary pneumomediastinum needs to be differentiated from SPM before treating the patient as the causes of secondary pneumomediastinum are different such as iatrogenic injury to the oesophagus due to endoscopy/blunt trauma or as the sequel of lung pathology. SPM is common in young males and in patients with asthmatic disorders. Although our patient was a young male, we could not find any underlying pathology causing SPM. The mechanism of SPM is not clearly understood. Macklin described in 1944 that SPM occurs due to the rupture of marginal alveoli due to increased alveolar pressure [3]. The air ascends along the mediastinum and reaches the subcutaneous tissue and forms subcutaneous emphysema. This kind of presentation is called Hamman's syndrome. Coughing, intense retching, illicit drug usage, asthma, childbirth are associated with Valsalva manoeuvre and hence considered as predisposing factors for SPM [5]. The most common symptom is retrosternal chest pain which might radiate to arms and jaw. Other symptoms include neck swelling, dysphagia, odynophagia which are relatively less common [4-6]. Our patient also had similar complaints of retrosternal chest pain and neck swelling. The general condition of the patients presenting with SPM is usually good with occasional tachypnoea. Clinically subcutaneous emphysema in the neck is the most common sign noticed in about 40%-100% of the population [2-4,7,8]. In 12% of the individuals, Hamman's sign can be elicited which is crunching sound occurring with every heartbeat [4]. Although our patient had neck swelling due to subcutaneous emphysema, the Hamman's sign was negative. The diagnosis in SPM is mainly through radiographic images. Chest X-ray with lateral and antero-posterior views is mandatory since small retrosternal pneumomediastinum can be missed in postero-anterior view [9]. However, in our patient pnemomediastinum and subcutaneous emphysema was evident on chest X-ray. Still, in 30% of the population, SPM can be missed in chest X-ray hence computed tomography (CT) neck and chest are considered the gold standard in the diagnosis [6,10,11]. CT with oral gastrograffin also helps in ruling out other causes of pneumomediastinum such as Boerhaave syndrome or pneumomediastinum following lung pathology [6]. We had performed high-resolution computed tomography (HRCT) of the thorax as well as contrast-enhanced computed tomography (CECT) of the thorax with oral gastrograffin, but we could not find any underlying pathology for the occurrence of subcutaneous emphysema and pneumomediastinum. Management is mostly conservative which includes adequate rest, oxygen by mask, and analgesia [4,5,7,8]. Patients respond well to this treatment and can be discharged within two to five days of the in-hospital stay [2,7,8]. Recurrences have been reported very rarely in the literature [5,12,13]. Our patient was managed conservatively with oxygen by mask, analgesics, and antibiotics. The patient was followed up for six months and there was no evidence of recurrence of the event.

## Conclusions

SPM is a rare benign condition which commonly occurs among young men. It should be considered as a differential diagnosis in young patients presenting with complaints of tachypnoea and subcutaneous emphysema. Diagnosis is mainly by imaging studies including chest X-ray and CECT of the thorax with water-soluble oral contrast to rule out other causes of pneumomediastinum. Patients usually respond well to conservative management and complete resolution can be expected in two to five days.
